# Impact of Vaccination on Cost and Course of Hospitalization Associated with COVID-19 Infection

**DOI:** 10.1017/ash.2022.364

**Published:** 2023-01-25

**Authors:** Selina T. Somani, Rachelle L. Firestone, Monica A. Donnelley, Luciano Sanchez, Chad Hatfield, Jeffrey Fine, Machelle D. Wilson, Jeremiah J. Duby

**Affiliations:** 1 Department of Pharmacy, University of California Davis Health, Sacramento, California; 2 Division of Biostatistics, University of California Davis, Sacramento, California

**Keywords:** COVID-19, vaccination, hospital length of stay, cost

## Abstract

**Objective:**

Examine the impact of vaccination status on hospital cost and course for patients admitted with COVID-19 infection.

**Design:**

Retrospective cohort study characterizing vaccinated and unvaccinated individuals hospitalized for COVID-19 between April 2021 to January 2022.

**Setting:**

Large academic medical center.

**Methods:**

Patients were included if they were greater than 18 years old, fully vaccinated or unvaccinated against COVID-19, and admitted for COVID-19 infection.

**Patients:**

437 consecutively admitted patients for COVID-19 infection met inclusion criteria. Of these, 79 were excluded for unknown or partial vaccination status, transfer from an outside hospital, or multiple COVID-19 related admissions.

**Results:**

Overall, 279 (77.9%) unvaccinated patients compared to 79 (22.1%) vaccinated patients were hospitalized with a diagnosis of COVID-19. Average length of stay was significantly lower in the vaccinated group (6.47 days versus 8.92 days, *P* = 0.03). Vaccinated patients experienced a 70.6% lower risk of ICU admission (OR = 0.29, 95% CI 0.12–0.71, *P* = 0.006). The unadjusted cost of hospitalization was not found to be statistically significant ($119,630 versus $191,146, *P* = 0.06). After adjusting for age and comorbidities, vaccinated patients experienced a 26% lower cost of hospitalization compared to unvaccinated patients (*P* = 0.004). Unvaccinated patients incurred a significantly higher cost of hospitalization per day ($29,425 vs $13,845 *P* < 0.0001). Unvaccinated patients (n = 118, 42.9%) were more likely than vaccinated patients (n = 16, 20.3%) to require high-flow oxygen or mechanical ventilation (OR = 2.95, 95% CI 1.62–5.38, *P* = 0.0004).

**Conclusion:**

Vaccinated patients experienced a lower cost of hospitalization after adjusting for age and comorbidities and shorter length of stay compared to unvaccinated patients admitted for COVID-19.

## Introduction

SARS-CoV-2 or COVID-19 is one of the most serious public health crises of our time. COVID-19 hospitalizations were a significant burden on healthcare systems nationwide. There was ongoing concern that the demand for resources created by the volume and acuity of COVID-19 patients would exceed available supplies.^1^ Scarce resources included hospital beds–intensive care unit (ICU) and general floor—and mechanical ventilators. Critical medications, from disease-modifying therapies (e.g., remdesivir, monoclonal antibodies) to neuromuscular-blocking agents, were also under continuous pressure and vulnerable to supply chain disruption.^1–3^

Patients who have been vaccinated against COVID-19 are less likely to become severely ill and require hospitalization.^4^ Full vaccination with mRNA vaccines (Pfizer BioNTech and Moderna) is approximately 80 - 90% effective in preventing hospitalization among adults aged ≥ 65 years.^5,6^ This trend is maintained in younger populations as well.^4^ Recent variants appear to spread faster, cause higher infection rates, and may cause infection even in fully vaccinated individuals. Even so, the risk of hospitalization, severe illness, and death remains much lower among vaccinated individuals.^7,8^ Current studies estimating the cost of hospitalization to the healthcare system based on vaccination status are theoretical in nature and relied upon data from administrative databases.^1,9–11^

This study aims to investigate the impact of vaccination on length of stay (LOS) and cost of COVID-19 hospitalization to the healthcare system.

## Materials and Methods

This was an observational, cohort study of patients admitted for COVID-19 infection to an academic medical center between April 2021 to January 2022. The study protocol was approved as exempt by the Institutional Review Board.

Patients were identified through the electronic medical record (Epic™). Patients were included if they were greater than 18 years old, fully vaccinated or unvaccinated against COVID-19, and admitted with a diagnosis of COVID-19. Patients who had unconfirmed or partial COVID-19 vaccination status, were transferred from an outside hospital, experienced multiple hospitalizations for COVID-19, or represented a vulnerable population (e.g., pregnant women or prisoners) were excluded. Full vaccination status for the study period was defined as two doses of either BNT162b2 mRNA (BioNTech-Pfizer) or Spikevax mRNA-1273 (Moderna) or one dose of Ad26.COV2-S vaccine (Janssen) at least two weeks prior to hospitalization.^12^ Partial vaccination was defined as one dose of either of the mRNA vaccines (Pfizer or Moderna) or less than two weeks from vector viral or second mRNA dose administration. Unvaccinated patients had not received a dose of a COVID-19 vaccine at the time of admission. The National Institute of Allergy and Infectious Diseases (NIAID) score was used to describe the intensity of respiratory support required based on disease severity upon admission and at the point of highest respiratory support.^13^ The full NIAID scoring definition can be found in Appendix 1. The Charlson Comorbidity Index was used to characterize patient’s baseline risk of complications and mortality. Cost data as total technical charges—which represented the total submitted to third party insurance or patient—was extracted by a financial analyst.

The primary endpoints were hospital length of stay and cost of hospitalization. Secondary endpoints included mortality, degree of respiratory support, and use of experimental disease modifying medications such as remdesivir, dexamethasone, tocilizumab, and baricitinib.^14^ Study data were collected and managed using REDCap electronic data capture tools.^15,16^

## Statistical Analysis

A prospective power analysis indicated that 32 patients in each group were required to detect a difference in length of stay of at least one day (standard deviation = 1 day) assuming an average length of stay of four days and an alpha < 0.05 and a beta of 0.8.

Descriptive statistics were used for baseline characteristics. Chi-squared test was used to compare categorical variables. Simple linear regression with analysis of covariance (ANCOVA) was used to determine and compare the slopes for cost of hospitalization. Kaplan-Meier with log-rank test was used to estimate the probability of hospitalization over time. A two-sided Student’s t-test was used for a comparison of means. Multivariable linear and logistic regression analyses were used to explore possible mediating effects and to evaluate differences in hospital LOS and cost, while controlling for age, comorbidities, and baseline NIAID score. SAS software version 9.4 (SAS Institute Inc., Cary, NC) for Windows was used for all analyses.

## Results

Of the 473 patients with a diagnosis of COVID-19 for the study period, 358 were included and 79 were excluded (Appendix 2). The primary reason for exclusion was unknown or partial vaccination status. The mean age was 57.1 ± 16.5 years, and the majority were male (51.4%), Table 1. The average body mass index (BMI) was 32.2 kg/m^2^ ± 11.9 kg/m^2^. In terms of the type of vaccine, 47 (59.5%), 22 (27.9%), and 10 (12.7%) received Pfizer BioNTech, Moderna, and Janssen respectively. There was a statistically significant difference in baseline comorbidities and oxygen requirements upon admission between vaccinated and unvaccinated patients (Table 1). Upon admission the risk of requiring intensive respiratory support (i.e., high-flow nasal cannula or mechanical ventilation) was substantially lower in the vaccinated group (OR 0.19, 95% CI 0.06 to 0.63, P = 0.006).

**Table 1.** Univariate Comparison of Baseline Characteristics

	Vaccinated (79)	Unvaccinated (279)	P-value
Age, years (mean ± SD)	66.5 (± 16.75)	54.5 (± 15.44)	<0.001
Sex (% female)	38 (48.1)	136 (48.8)	0.92
(% male)	41 (51.9)	143 (51.3)	
Charlson Comorbidity Index	5.46 (± 3.02)	2.27 (± 2.39)	<0.0001
Ethnicity:			
Hispanic or Latino	20 (25.3)	77 (27.9)	0.65
Non-Hispanic or Latino	59 (74.7)	199 (72.1)	
Race			
White	37 (46.8)	109 (39.5)	0.67
Black	14 (17.7)	50 (18.1)	
Asian	5 (6.3)	19 (6.9)	
Other	23 (29.1)	98 (35.5)	
BMI^a^ (kg/m^2^)	31.3 (± 7.87)	32.5 (± 12.79)	0.32
Type of vaccine received			
Pfizer	47 (59.5)	–	
Moderna	22 (27.9)	–	
Jansen	10 (12.7)	–	
Baseline NIAID Score^b^			
NIAID 4	42 (53.2)	131 (47.0)	0.03
NIAID 5	34 (43.0)	100 (35.8)	
NIAID 6	3 (3.8)	47 (16.9)	
NIAID 7	0	1 (0.36)	

a Body Mass Index (BMI).

b National Institute of Allergy and Infectious Disease score (NIAID) 4 = hospitalization without supplemental oxygen, 5 = supplemental oxygen, 6 = noninvasive ventilation or high-flow oxygen, 7 = mechanical ventilation, 8 = death due to COVID-19 complications.

**Figure 1.**
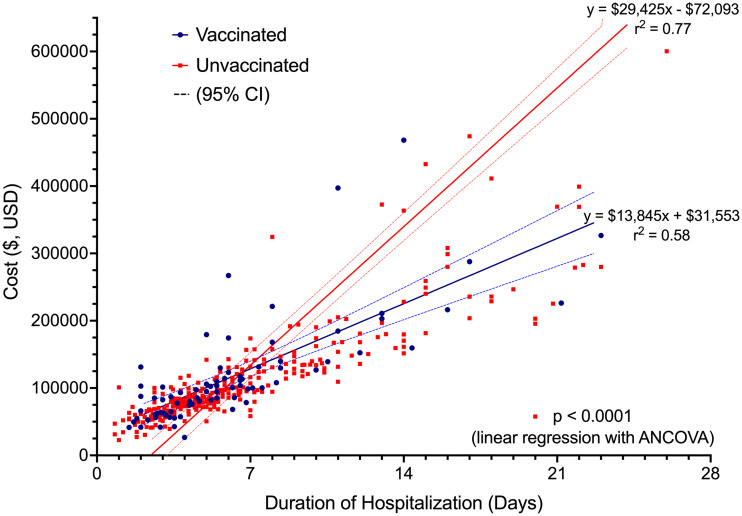
Cost of hospitalization and length of stay based on vaccination status.

For the primary outcome, length of stay was significantly lower in the vaccinated group compared to the unvaccinated group, 6.47 ± 4.76 days versus 8.92 ± 9.80 days, respectively, P = 0.03, (Table 2). The unadjusted difference in cost of hospitalization was not statistically significant between the vaccinated group compared to the unvaccinated group, ($119,630 ± $78,833 versus $191,146 ± $328,233, P = 0.06, Table 2). Unvaccinated patients incurred a significantly higher cost of hospitalization per day ($29,425 vs $13,845 P < 0.0001, Figure 1).

**Table 2.** Univariate Comparison of Clinical Outcomes and Interventions Received During Hospitalization

	Vaccinated (79)	Unvaccinated (279)	P-value
**Primary endpoints**			
Length of Stay, days (mean ± SD)	6.47 ± 4.76	8.92 ± 9.80	0.03
Cost of hospitalization, $ (mean ± SD)	119,629.5 ± 78,833.08	191,146.4 ± 328,233.7	0.06
**Secondary endpoints**			
ICU Length of Stay, days (mean ± SD)	3.31 ± 3.42	7.13 ± 11.40	0.07
Mechanical Ventilation^a^ (%)	2 (2.53)	20 (7.17)	–
Mechanical Ventilation Duration, days (mean ± SD)	3.01 ± 2.75	17.81 ± 14.00	0.003
Mortality	3 (3.8)	9 (3.2)	0.80
Highest NIAID^b^ score reached			
NIAID 4	11 (13.9)	22 (7.9)	0.002
NIAID 5	52 (65.8)	135 (48.4)	
NIAID 6	13 (16.5)	98 (35.1)	
NIAID 7	0	15 (5.4)	
NIAID 8	3 (3.8)	9 (3.2)	
Medication Received			
Remdesivir	65 (82.3)	257 (92.1)	0.01
Dexamethasone	48 (60.8)	236 (84.6)	<0.001
Tocilizumab	2 (2.5)	30 (10.8)	0.02
Baricitinib	3 (3.8)	35 (12.5)	0.03

a Sample of unvaccinated patients that required mechanical ventilation was too small for responsible statistical comparison.

b National Institute of Allergy and Infectious Disease score (NIAID).

**Figure 2.**
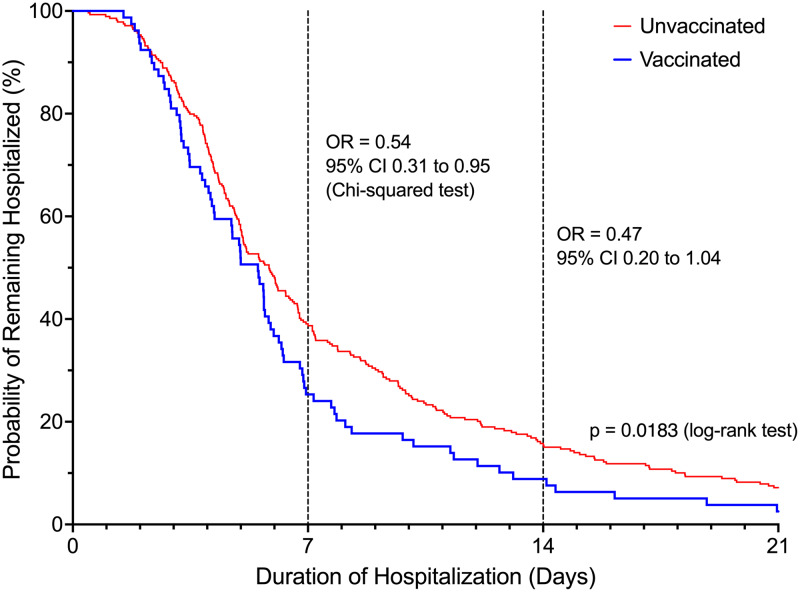
Probability of remaining hospitalized based on vaccination status. Odds ratios were reported for hospital day 7 and day 14.

**Figure 3.**
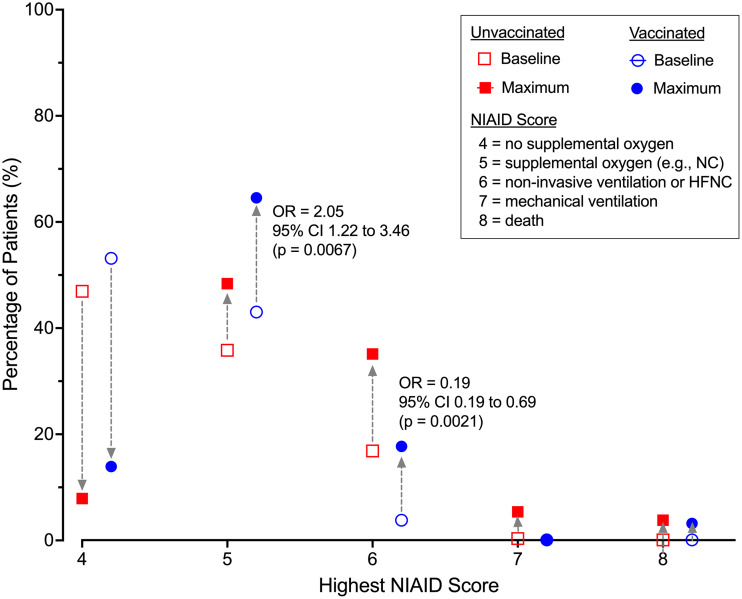
Highest level of respiratory support needed based on vaccination status. Odds ratios calculated compare vaccinated to unvaccinated patients. NIAID- National Institute of Allergy and Infectious Diseases.

Patients in the vaccinated group were 46.2% less likely to be hospitalized beyond 7 days (OR = 0.54, 95% CI 0.31 – 0.95, P = 0.03) compared to unvaccinated patients (Figure 2). The difference in the risk of prolonged hospitalization for vaccinated patients, defined as greater than 14 days, was not statistically significant (OR = 0.47, 95% 0.20-1.04, P = 0.08). Vaccinated patients experienced a 70.6% lower risk of ICU admission (OR = 0.29, 95% CI 0.12 to 0.71, P = 0.006). There was no difference in ICU LOS between the vaccinated (3.31 ± 3.42 days) and unvaccinated patients (7.13 ± 11.40 days, P = 0.07) in the subgroup who were admitted to the ICU. Overall, there was a statistically significant difference in the highest NIAID scores (P = 0.002) reached over the course of hospitalization comparing vaccinated to unvaccinated patients (Table 2). Supplemental oxygen with a nasal cannula was the highest intensity of respiratory support needed for most (n = 63, 79.7%) vaccinated patients. Unvaccinated patients (n = 118, 42.9%) were substantially more likely than vaccinated patients (n = 16, 20.3%) to require high-flow oxygen or mechanical ventilation (OR = 2.95, 95% CI 1.62 – 5.38, P = 0.0004, Figure 3). The proportion of mechanical ventilation was lower in the vaccinated group (2.5%) compared to the unvaccinated group (7.2%) although the difference did not achieve statistical significance (OR = 0.34, 95% CI 0.08 – 1.47, P = 0.15). However, the difference in mean duration of mechanical ventilation between vaccinated (3.01 ± 2.75 days) and unvaccinated (17.81 ± 14.00 days) patients was statistically significant (P = 0.003). The risk of requiring costly experimental pharmacotherapy was substantially lower in vaccinated patients (OR = 0.22, 95% CI 0.09-0.57, P = 0.002).

Age and comorbidities mediated the effect of vaccination status on length of stay. The covariate analysis demonstrated a 32% lower length of stay compared to those who were unvaccinated, after adjusting for age and comorbidities (P = 0.0004, Appendix 3). Patients who were vaccinated had a 25% lower length of stay compared to those who were unvaccinated, after adjusting for age, comorbidities, and baseline NIAID (P = 0.004, Appendix 3). Additionally, patients who were vaccinated had a 26% lower cost of hospitalization compared to those who were not vaccinated after adjusting for age and comorbidities (P = 0.004, Appendix 3). Baseline NIAID score mediated the effect of vaccination status on cost. Vaccinated patients had a 19% lower cost of hospitalization compared to unvaccinated patients after adjusting for age, comorbidities, and baseline NIAID; P = 0.04, Appendix 3).

## Discussion

To our knowledge, this is the first study in the United States to report the impact of vaccination on LOS and actual cost of COVID-19 hospitalization to the healthcare system. Patients who were fully vaccinated for COVID-19 experienced a shorter hospital LOS, lower rate of ICU admission, and less severe respiratory failure. Additionally, after adjusting for age and comorbidities, vaccinated patients incurred a lower cost of hospitalization. No differences were observed between unvaccinated and vaccinated patients for ICU LOS or mortality. This may be explained by the exclusion of patients who were transferred from an outside hospital or who had multiple admissions for COVID-19. By every clinical measure, there was no advantage to remaining unvaccinated over the course of hospitalization for COVID-19.

Patients who were unvaccinated were more likely to qualify for and receive dexamethasone and experimental medication therapies (i.e., remdesivir, tocilizumab, baricitinib) than vaccinated patients (Table 1, 2). Tocilizumab and baricitinib were administered under emergency use authorization (EUA) for COVID-19 at the time of the study. However, there were significant barriers to the use of these medication therapies including, high cost, supply chain shortages, and ethical concerns (i.e., competing demand for CAR-T rescue).^17,18^ The cost to the health system for full course remdesivir, tocilizumab, and baricitinib were $2,145, $3,458, and $2,512, respectively at the time of treatment. For the study period, only a small proportion of patients received tocilizumab or baricitinib due to supply constraints. These costs may appear relatively insignificant compared to the overall outlay of hospitalization. However, an additional cost of $383,916 would have been incurred by unvaccinated patients if ample supply of tocilizumab had been available for those who qualified for treatment upon admission.^19,20^ Furthermore, it is impossible to infer the benefit of these therapies on length of stay, which was likely the primary driver of cost.

Strengths of this study include the novelty of the study question and use of actual cost data to describe hospitalization. Baseline characteristics were generally well matched between cohorts with the only significant differences being age, comorbidities, and baseline NIAID score. Extrapolation of the study findings are limited by the single-center design, and the experience of COVID-19 is likely unique to each region and healthcare system. The relatively small sample size is another consideration; however, the study achieved adequate power for the primary outcomes. The retrospective design may have resulted in additional, unknown effects on the study outcomes. However, ethical considerations—e.g., assigning patients to vaccinated or unvaccinated groups—would obviously limit the feasibility of a prospective study design. As a tertiary and quaternary referral center, a significant proportion of patients with severe complications of COVID-19 infection were transferred from referring hospitals. These patients were all excluded from study analysis due to the inability to determine the costs of hospitalization prior to transfer. This likely mitigated the primary outcomes in favor of the unvaccinated cohort of patients. Additionally, the investigators did not compare complications between different intervals post vaccination or examine the effect of waning immunity. No test of bias was performed in the application or usage of disease modifying therapies between vaccinated and unvaccinated patients. However, rubrics based on risk factors for COVID-19 progression were utilized to provide disease modifying agents to patients. Lastly, the investigators did not collect data regarding specific COVID-19 variants.

Vaccinated patients experienced a shorter length of stay and a lower cost of hospitalization compared to unvaccinated patients admitted for COVID-19 infection.

## Supplementary material

For supplementary material accompanying this paper visit https://doi.org/10.1017/ash.2022.364. click here to view supplementary material
