# Supplementary material for: Impact of Vaccination on Cost and Course of Hospitalization Associated with COVID-19 Infection
Source: Antimicrob Steward Healthc Epidemiol. 2023 Jan 25;3(1):e19. doi: 10.1017/ash.2022.364 (PMC9879923; doi:10.1017/ash.2022.364)
Supplement: Supplementary file 1 [file S2732494X22003643sup001.docx]

**Appendix 1. NIAID Score Definition**

| **NIAID Score** | **Description** |
| --- | --- |
| **4** | hospitalization and ongoing medical care without supplemental oxygen |
| **5** | supplemental oxygen (e.g., nasal cannula) |
| **6** | noninvasive ventilation or high-flow oxygen device |
| **7** | mechanical ventilation |
| **8** | death due to complications of COVID-19 |

*Hospitalization was indicated for all NIAID scores greater than 5

**Appendix 2. Consort Diagram**


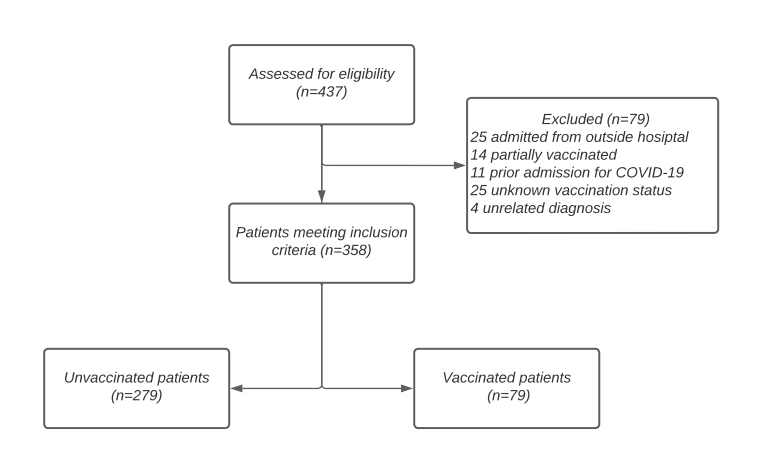


**Appendix 3. Regression Analysis Models**

Model 1: Does vaccination status predict LOS after adjusting for age and Charlson Comorbidity Index

| **Dependent** | **Parameter** | **Estimate** | **StdErr** | **tValue** | **Probt** | **Back transformed estimate** |
| --- | --- | --- | --- | --- | --- | --- |
| log_los | Intercept | 1.53982 | 0.1673 | 9.2 | <.0001 | 4.66375 |
| log_los | Vaccination Status Full | -0.3836 | 0.10673 | -3.59 | 0.0004 | 0.68138 |
| log_los | Vaccination Status None | 0 | . | . | . | 1 |
| log_los | Charlson Comorbidity Index | 0.0491 | 0.02191 | 2.24 | 0.0256 | 1.05032 |
| log_los | Age | 0.00354 | 0.00354 | 1 | 0.3177 | 1.00355 |

Those that are vaccinated have a 32% (1-0.68=0.32) lower LOS compared to those that are not vaccinated after adjusting for age and Charlson Comorbidity Index (*P*=0.0004).

Model 2: Does vaccination status predict LOS after adjusting for age, Charlson Comorbidity Index, and baseline NIAID

| **Dependent** | **Parameter** | **Estimate** | **StdErr** | **tValue** | **Probt** | **Back transformed estimate** |
| --- | --- | --- | --- | --- | --- | --- |
| log_los | Intercept | 2.5451 | 0.70412 | 3.61 | 0.0003 | 12.7445 |
| log_los | Vaccination Status Full | -0.2916 | 0.10141 | -2.88 | 0.0043 | 0.7471 |
| log_los | Vaccination Status None | 0 | . | . | . | . |
| log_los | Charlson Comorbidity Index | 0.04725 | 0.02065 | 2.29 | 0.0227 | 1.0484 |
| log_los | Age | 0.00286 | 0.00334 | 0.85 | 0.3939 | 1.0029 |
| log_los | Baseline_NIAID NIAID 4 | -1.2071 | 0.70243 | -1.72 | 0.0866 | 0.2991 |
| log_los | Baseline_NIAID NIAID 5 | -0.888 | 0.70385 | -1.26 | 0.2079 | 0.4115 |
| log_los | Baseline_NIAID NIAID 6 | -0.4673 | 0.70743 | -0.66 | 0.5094 | 0.6267 |
| log_los | Baseline_NIAID NIAID 7 | 0 | . | . | . | . |

Those that are vaccinated have a 25% (1-0.75=0.25) lower LOS compared to those that are not vaccinated after adjusting for age, Charlson Comorbidity Index, and baseline NIAID (*P*=0.004).

Model 3: Does vaccination status predict cost after adjusting for age and Charlson Comorbidity Index

| **Dependent** | **Parameter** | **Estimate** | **StdErr** | **tValue** | **Probt** | **Back transformed estimate** |
| --- | --- | --- | --- | --- | --- | --- |
| log_cost | Intercept | 11.4275 | 0.16578 | 68.93 | <.0001 | 91814.5 |
| log_cost | Vaccination Status Full | -0.3074 | 0.10577 | -2.91 | 0.0039 | 0.74 |
| log_cost | Vaccination Status None | 0 | . | . | . | 1 |
| log_cost | Charlson Comorbidity Index | 0.03943 | 0.02171 | 1.82 | 0.0702 | 1.04 |
| log_cost | Age | 0.00295 | 0.00351 | 0.84 | 0.4004 | 1 |

Those that are vaccinated have a 26% (1-0.74=0.26) lower COST compared to those that are not vaccinated after adjusting for age and Charlson Comorbidity Index (*P*=0.004).

Model 4: Does vaccination status predict cost after adjusting for age, Charlson Comorbidity Index, and baseline NIAID

| **Dependent** | **Parameter** | **Estimate** | **StdErr** | **tValue** | **Probt** | **Back transformed estimate** |
| --- | --- | --- | --- | --- | --- | --- |
| log_cost | Intercept | 12.7391 | 0.68407 | 18.62 | <.0001 | 340813 |
| log_cost | Vaccination Status Full | -0.2049 | 0.09853 | -2.08 | 0.0383 | 0.81 |
| log_cost | Vaccination Status None | 0 | . | . | . | . |
| log_cost | Charlson Comorbidity Index | 0.03722 | 0.02006 | 1.86 | 0.0644 | 1.04 |
| log_cost | Age | 0.00226 | 0.00325 | 0.7 | 0.4875 | 1 |
| log_cost | Baseline_NIAID NIAID 4 | -1.5405 | 0.68244 | -2.26 | 0.0246 | 0.21 |
| log_cost | Baseline_NIAID NIAID 5 | -1.1841 | 0.68382 | -1.73 | 0.0842 | 0.31 |
| log_cost | Baseline_NIAID NIAID 6 | -0.7188 | 0.68729 | -1.05 | 0.2964 | 0.49 |
| log_cost | Baseline_NIAID NIAID 7 | 0 | . | . | . | . |

Those that are vaccinated have a 19% (1-0.81=0.19) lower COST compared to those that are not vaccinated after adjusting for age, Charlson Comorbidity Index, and baseline NIAID (*P*=0.04).
